# Prostate Stereotactic Body Radiation Therapy With Halcyon 2.0: Treatment Plans Comparison Based on RTOG 0938 Protocol

**DOI:** 10.7759/cureus.11660

**Published:** 2020-11-23

**Authors:** Yücel Altundal, Fulya Cifter, Guangwei Mu, James Lee, Elisa J Wu, Vincent Yeung, Alan Katz

**Affiliations:** 1 Medical Physics, Flushing Radiation Oncology Services, Flushing, USA; 2 Medical Physics, Precess Medical, Short Hills, USA; 3 Radiation Oncology, Flushing Radiation Oncology Services, Flushing, USA; 4 Radiation Oncology, St. Francis Hospital, Roslyn, USA

**Keywords:** radiotherapy, sbrt, halcyon, rtog 0938, cyberknife

## Abstract

Purpose

The aim of this study is to investigate the feasibility of prostate stereotactic body radiation therapy treatment with a newly developed Varian Halcyon^TM^ 2.0 machine by comparing radiotherapy plans with previously delivered CyberKnife G4 plans created with the previous version of CyberKnife Treatment Planning System Multiplan 4.6.1.

Methods

Fifteen previously treated prostate stereotactic body radiation therapy treatment CyberKnife plans were re-planned retrospectively according to the Radiation Therapy Oncology Group 0938 protocol on a Halcyon^TM^ 2.0 machine with a prescription of 3625 cGy in five fractions.

Results

All re-plans on a Halcyon^TM^ 2.0 were able to meet the Radiation Therapy Oncology Group 0938 protocol goals and constraints. The re-plans decreased the maximum dose to skin and urethra, mean doses to the bladder and rectum, and also improve the conformity index and the Planning Target Volume coverage. However, D1cc to the rectum, D1cc and D10% to the bladder increased with no statistically significant differences (p > 0.05) with the re-plans.

Conclusion

The Halcyon^TM^ 2.0 can generate stereotactic body radiation therapy treatment prostate plans created based on the Radiation Therapy Oncology Group 0938 protocol by delivering adequate coverage to the target while sparing healthy tissues.

## Introduction

Hypo-fractionated stereotactic body radiation therapy (SBRT) is a method to deliver large and precise doses of radiation externally to an extracranial target within the body in a few fractions [1]. Administration of highly conformal radiation with this technique to small, well defined, and well-targeted tumors spares the surrounding healthy tissue by rapid dose fall-off. For prostate cancer patients, the conventional external beam therapy lasts about eight to nine weeks, up to about 80 Gy [2]. SBRT for prostate treatment uses higher daily doses in fewer fractions, which is radiobiologically beneficial due to the low alpha/beta ratio of prostate cancer [3].

The long-term good outcomes with minimal toxicity of prostate cancer SBRT treatment using the CyberKnife System (Accuray Incorporated, Sunnyvale, CA, USA) has been discussed in several papers [4-9]. This system uses an image-guided linear accelerator mounted on a robotic arm to deliver external beam radiation [10]. The real-time image guidance with five or six-degrees-of-freedom couch provides precise delivery of high radiation doses to the target by tracking fiducials implanted in the prostate [11,12], and correcting the position errors in near real time by manipulating the treatment head using the highly precise industrial robot attached to the treatment head.

Meanwhile, a newly developed closed system linear accelerator (LINAC) with a 100-cm diameter bore size, Halcyon^TM^ (Varian Medical System, Palo Alto, CA, USA), was introduced to simplify the treatment workflow and speed up the delivery of the radiation. Halcyon^TM^ 2.0 with a kV cone-beam computed tomography (CBCT) delivers a single 6MV flattening filter-free (FFF) beam with a double stack multi-leaf collimator (MLC), SX2. The width of SX2 leaves is 1 cm at the isocenter with a 0.5-cm offset at the isocenter to minimize the leakage between the proximal and distal leaves. This double stack MLC design renders an effective leaf resolution of 5 mm at the isocenter. The SX2 collimator allows both the proximal and distal leaves to modulate the beam. The increased gantry speed is 4 rpm, the maximum leaf speed is 5 cm/s with a dose rate of 800 MU/min at dmax of 1.3 cm for a 100-cm SSD setup [13,14].

This study aims to investigate the feasibility of prostate SBRT treatment with a Halcyon^TM^ 2.0 linac. For this purpose, 15 SBRT prostate plans previously delivered at a CyberKnife (CK) were compared with plans recreated for a Halcyon^TM^ 2.0. The CyberKnife plans were generated based on the Radiation Therapy Oncology Group (RTOG) 0938 protocol prescription, goals, and constraints [15]. The plans were compared based on the RTOG 0938 protocol. In addition to those goals and constraints, mean doses of planning target volume (PTV), rectum, and bladder; minimum dose to prostate; PTV and prostate dose coverages; conformity index (CI), Paddick conformity index (PCI) and Homogeneity index (HI) were also compared [16,17].

## Materials and methods

Patient selection

Fifteen prostate cancer patients previously treated at the Flushing Radiation Oncology Center (FROS) between August 2019 and May 2020 with a total prescription of 3625 cGy were selected randomly. These patients were treated with a CyberKnife G4 unit in five fractions according to the RTOG 0938 protocol.

Imaging, delineation, and SBRT treatment plan requirements

Computed tomography (CT) simulations were performed with a 1-mm scan slice thickness in a feet-first supine position and then fused with 2 mm T2 magnetic resonance (MR) images. Fusions were performed by matching four fiducials implanted into the prostate. The corresponding physicians contoured prostate (clinical target volume), rectum, bladder, urethra, penile bulb, and bowels. Planning target volumes were created by extending the clinical target volume (CTV) 3 mm posteriorly, and 5 mm in all other directions. The planner contoured femoral heads and 15 mm thick skin.

Treatment planning

A CyberKnife G4 Image-Guided Robotic Stereotactic Radiosurgery System unit with a variable IRIS was used to treat those patients. The IRIS circular collimator delivers cone-shaped beams with a diameter of 5 to 60 mm nominal size at a SAD of 80 cm. Multiplan 4.6.1 TPS with Ray-Tracing calculation method and sequential optimization algorithm was used to generate plans. All plans were delivered with the IRIS collimator. The prescription dose was prescribed to 78-81% isodose lines to get coverage of 93.99-95.44% of the PTV volume. The minimum and maximum MU sets were 25 and 770 per beam, respectively. A computer-controlled five degrees of freedom (DoF) treatment couch was used to position the patient, the yaw rotational correction being the left out. The rotational (yaw) corrections were corrected manually in the room by therapists before start of the treatment delivery. The gold fiducials implanted inside the prostate were used for target localization and tracked during the treatments with two orthogonal kilo-voltage X-ray and detector systems by imaging in about every 30-40 seconds. The linac delivered 6MV flattening filter-free (FFF) Photon Energy with a maximum dose rate of 1000 MU/min.

The Halcyon^TM^ plans were generated with the Eclipse TPS version of 15.6.06. The inverse planning technique with the photon optimizer (PO) version of 15.6.06 was used, and dose calculations were performed with the analytical anisotropic algorithm (AAA) version of 15.6.06. Three or four full arcs with fine resolution (1.25 mm) were used to generate coplanar volumetric modulated arc therapy (VMAT) plans. Eight of the plans were generated with three full arcs with automatically generated 285, 345, and 45 degrees of collimator angles. Four full arcs with automatically generated 281, 326, 11, and 56 degrees of collimator angles were used for the rest of the plans. In addition to RTOG protocol (Table 1), the planner aimed to have at least 99% of the volume of the prostate (CTV) to get 100% of the prescription dose (3625 cGy) and minimize the doses to the healthy tissues as much as possible. Once plans were satisfied with the desired coverages and constraints, no more adjustments were made.

The RTOG 0938

The RTOG 0938 goals and constraints given in Table 1 of CK and Halcyon plans were compared [15].

**Table 1:** The RTOG 0938 goals and constraints.

Organ	Volume	Dosimetry parameters for 5 fraction arm and with all delivery devices except CyberKnife	Dosimetry parameters for 5 fraction arm and treatment with CyberKnife
PTV	Max point dose	≤38.78 Gy, 107% of prescription	≤43.5 Gy, 120% of prescription
	Min dose received by 95% of PTV	≥36.25 Gy, 100% of prescription	SAME
	Min dose received by PTV	≥34.4 Gy, 95% of prescription	SAME
Rectum	Max point dose (1cc)	≤38.06 Gy, 105% of prescription	SAME
	Less than 3 cc	<34.4 Gy, 95% of prescription	SAME
	10% rectum	≤32.625 Gy, 90% of prescription	SAME
	20% rectum	≤29 Gy, 80% of prescription	SAME
	50% rectum	≤18.125 Gy, 50% of prescription	SAME
Bladder	Max point dose (1cc)	≤38.06 Gy, 105% of prescription	SAME
	10% bladder	≤32.625 Gy, 90% of prescription	SAME
	50% bladder	≤18.125 Gy, 50% of prescription	SAME
Penile Bulb (recommended)	Max point dose	No more than 100% of prescription	SAME
	Less than 3cc	20 Gy, 54% of prescription	SAME
Fem Heads_L (recommended)	Max point dose	30 Gy, 81% of prescription	SAME
	Less than 10cc	20 Gy, 54% of prescription	SAME
Fem Heads_R (recommended)	Max point dose	30 Gy, 81% of prescription	SAME
	Less than 10cc	20 Gy, 54% of prescription	SAME
Skin (recommended)	Max point dose	30 Gy, 81% of prescription	SAME
	Less than 10cc	20 Gy, 54% of prescription	SAME
Urethra dose	Max point dose	≤38.78 Gy, 107% of prescription	SAME

Other dosimetric parameters

In addition to RTOG 0938 goals and constraints, PTV mean dose, the percentages of PTV and CTV volumes that receive prescription dose (coverage), the minimum prostate dose, rectum mean dose, bladder mean dose, CI, PCI and HI of the CK and Halcyon plans were compared. The CI, PCI and HI are defined as follows:

\\CI=\frac{\textrm{Volume Covered by Prescribtion Isodose}}{\textrm{Planning Target Volume} } \\ PCI=\frac{\left (\textrm{Volume of the Target Covered by the Prescription Isodose}\right )^{2}}{\textrm{(Planning Target Volume})\cdot(\textrm{Volume Covered by Prescription Isodose})} \\ HI=\frac{\textrm{Maximum Dose}}{\textrm{Prescription Dose}} \\

Conformity index given by the first equation was defined by RTOG radiosurgery guidelines to evaluate how well a target is covered by the volume of the prescription isodose lines [18]. However, CI does not take into account the shape and the location of the prescription isodose line volume. To account this Paddick conformity index (second equation) was proposed by Paddick [16] to evaluate the degree of overlapping of the target volume and the prescription isodose line volume. The values for both the CI and PCI close to 1.0 are considered more conformal. On the other hand, the homogeneity index is useful to evaluate the homogeneity of dose distribution within the target. A value of 1.0 indicates a perfect homogeneous plan.

Statistical analysis

The small sample size nonparametric Wilcoxon signed-rank test was used to analyze all the data with a p-value less than 0.05 considered to be statistically significant (IBM® SPSS® Statistics Subscription, IBM Corporation, Armonk, NY).

Plan quality assurance (QA)

The pre-treatment patient plan quality assurance (QA) of each plan was performed by using Varian Portal Dosimetry (PD) with a 2%/2 mm gamma index passing metric and a 10% threshold.

## Results

RTOG 0938

The average values of the RTOG 0938 goals and constraints with standard deviations and p-values of 15 plans are given in Table 2. All the Halcyon VMAT plans can satisfy the goals and constraints of the RTOG 0938 protocol hence the average values. However, the averaged CK plans fail to satisfy any of the PTV goals and the urethra constraint. There are statistically significant differences in the goals and the constraints of the protocol between the CK and Halcyon plans except for D1cc, D3cc, and D50% of the rectum, D1cc and D10% of the bladder constraints.

**Table 2:** Average values, standard deviations and p-values of CyberKnife and Halcyon plans. PTV: Planning target volume

		CK		Halcyon		
Organ	Volume	Average (cGy)	STD (cGy)	Average (cGy)	STD (cGy)	p-value
Prostate (PTV)	Max point dose	4547	46	3843	22	<0.001
	Min dose received by 95% of PTV	3618	12	3635	13	0.006
	Min dose received by PTV	2896	186	3479	30	<0.001
Rectum	Max point dose (1cc)	3566	74	3609	66	0.061
	Less than 3 cc	3298	94	3273	128	0.495
	10% rectum	3118	131	3041	145	0.017
	20% rectum	2595	210	2419	141	0.036
	50% rectum	1527	228	1416	216	0.100
Bladder	Max point dose (1cc)	3670	145	3695	42	0.670
	10% bladder	2716	463	2789	416	0.334
	50% bladder	1217	421	909	502	0.001
Penile Bulb	Max point dose	2832	616	2522	802	0.004
	Less than 3 cc	473	578	214	253	N/A
Fem Heads_L	Max point dose	938	285	1169	238	0.005
	Less than 10 cc	549	219	829	208	0.003
Fem Heads_R	Max point dose	1482	248	1212	233	0.020
	Less than 10 cc	1105	214	890	216	0.017
Skin	Max point dose	1849	45	1518	138	<0.001
	Less than 10 cc	1452	105	1205	117	<0.001
Urethra dose	Max point dose	4158	109	3793	22	<0.001
The significance level is 0.05.						

Other dosimetric parameters

**Figure 1:**
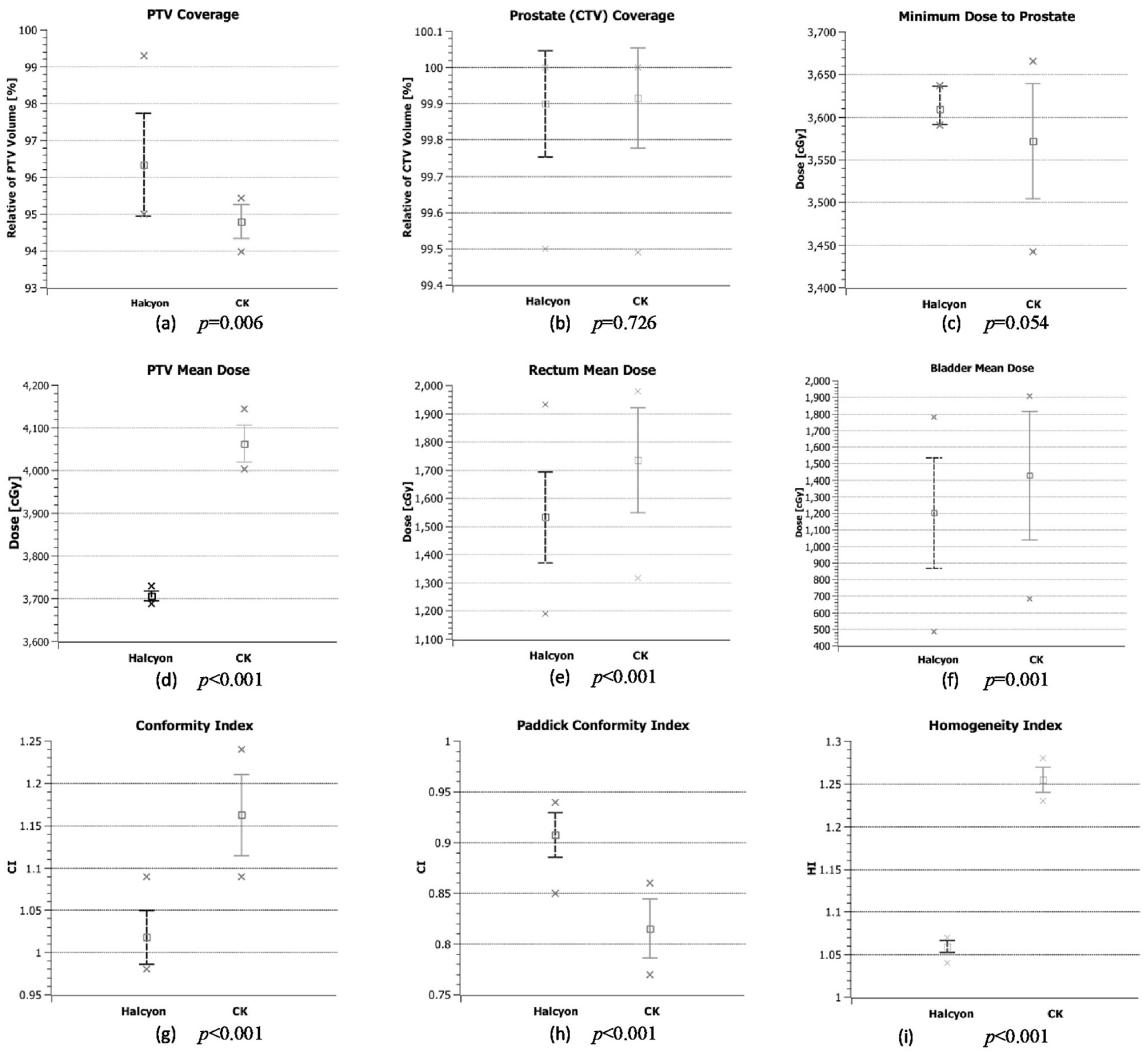
Average values of dosimetric parameters. Average values, one standard deviation (bars), maximum and minimum (x) values of (a) Planning Target Volume (PTV) coverage, (b) CTV coverage, (c) Minimum point dose to Clinical Target Volume (CTV), (d) Planning Target Volume (PTV) mean dose, (e) Rectum mean dose, (f) Bladder mean dose, (g) Conformity Index, (h) Paddick Conformity Index and (i) Homogeneity Index of CyberKnife (CK) and Halcyon plans.

Figure 1 shows the average values of coverages of PTV and CTV; minimum dose to CTV; mean doses of PTV, rectum, and bladder; conformity index, Paddick conformity index and homogeneity index with one standard deviation, maximum and minimum values of Halcyon and CK plans. There are no statistically significant differences only between the prostate dose coverage and prostate minimum dose among those parameters.

The prescription dose coverage (volume getting 100% of the prescription dose) of the PTV and CTV are 96.34 ± 0.36% (mean ± standard error) and 99.90 ± 0.04%, respectively; the average mean doses of the PTV, bladder and rectum are 3706 ± 3 cGy, 1203 ± 86 cGy and 1533 ± 42 cGy, respectively; and the CI, PCI and HI are 1.02 ± 0.01, 0.91 ± 0.01, 1.06 ± 0.01, respectively, for the Halcyon VMAT plans.

Monitor unit and delivery time for Halcyon plans

**Figure 2:**
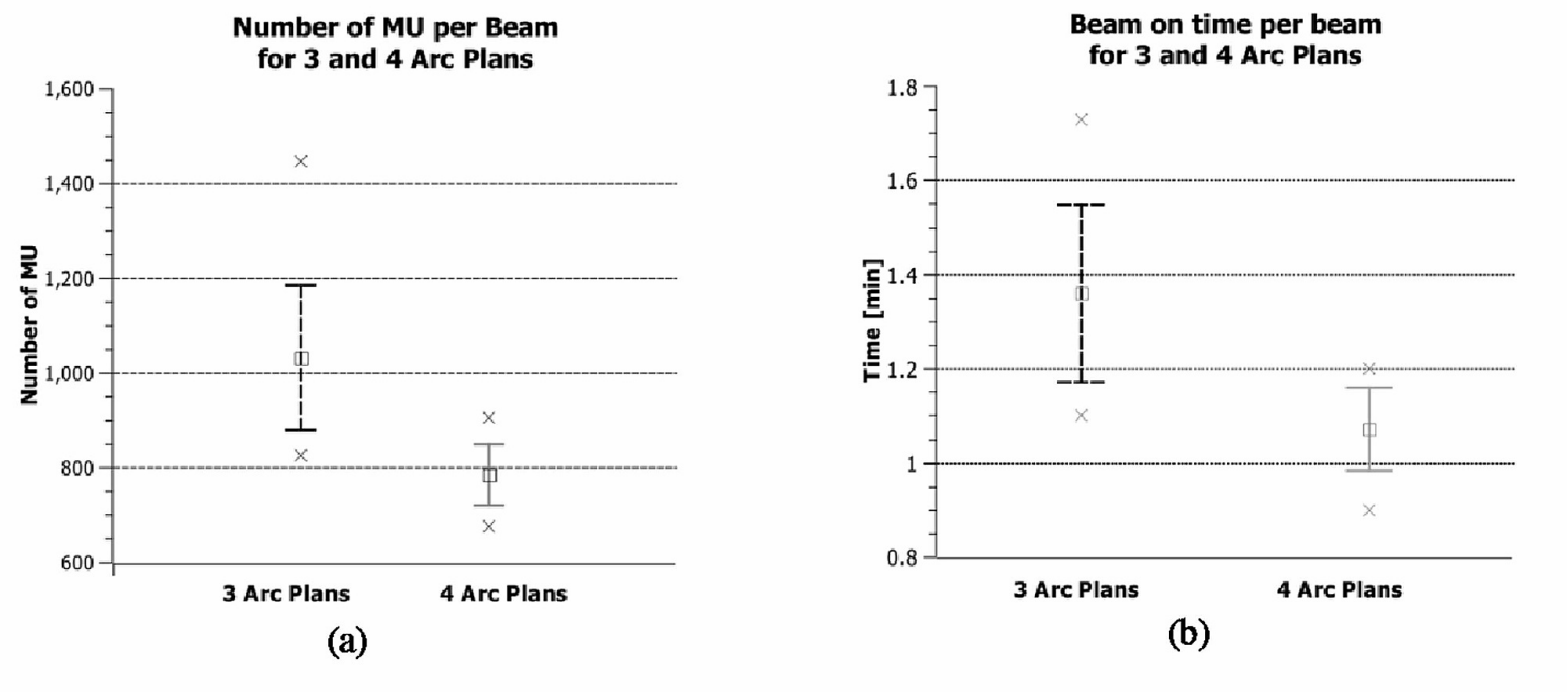
Monitor unit and delivery time for Halcyon plans. Average values, one standard deviation, maximum and minimum values of (a) MU per beam and (b) beam-on time per beam for three and four arc Halcyon plans.

Figure 2a and Figure 2b show the average values of monitor units (MU), and QA beam-on time with one standard deviation, maximum and minimum values for three and four arc VMAT Halcyon plans, respectively. For 3-Arc plans, the average total monitor unit and QA beam-on time are 3093.9 ± 134.9 MU and 4.12 ± 0.18 min, respectively, for one fraction. For 4-Arc plans, the average total monitor unit and QA beam-on time are 3143.9 ± 88.5 MU and 4.19 ± 0.12 min, respectively. The monitor unit and beam-on time per beam for 3-Arc plans are 1031.3 ± 31.2 MU and 1.37 ± 0.04 min, respectively. For 4-Arc plans, the monitor unit and beam-on time per beam are 786.0 ± 12.3 MU and 1.05 ± 0.02 min, respectively. Owing to the high number of small segments in CK, the high number of MU’s was a limitation to compare (i.e. 36,333 ± 455 MU vs 3143.9 ± 88.5 MU).

Plan QAs

The QA pass rate of each beam is 99.75 ± 0.08 with a 2%/2mm gamma index and a 10% threshold.

## Discussion

Dosimetric comparison of CK and Halcyon plans

**Figure 3:**
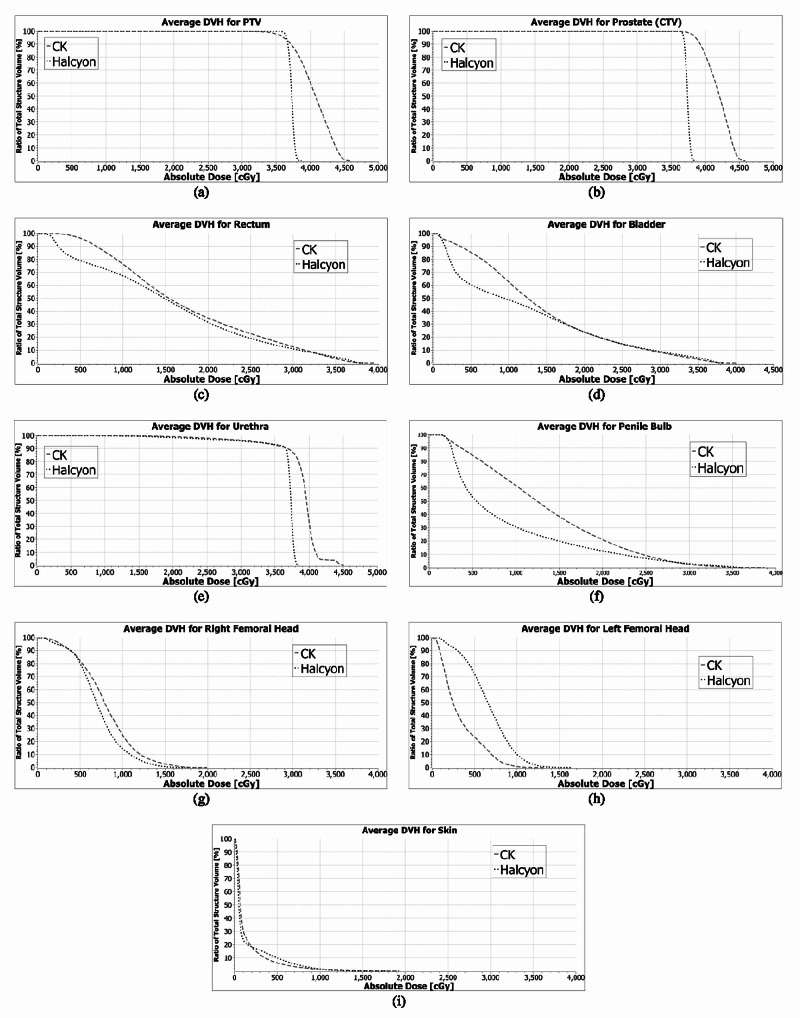
Average dose volume histograms of CK and Halcyon plans.

**Figure 4:**
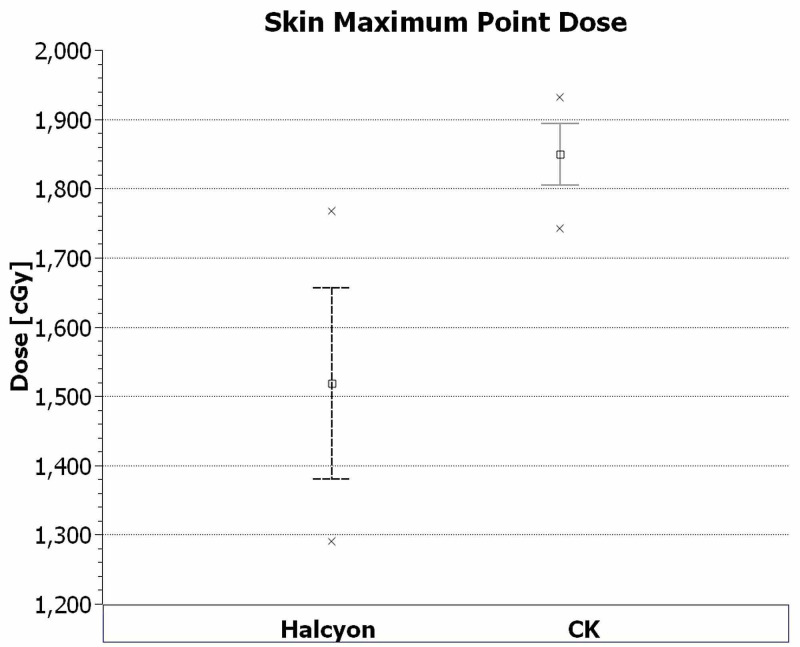
Average maximum dose to skin.

**Figure 5:**
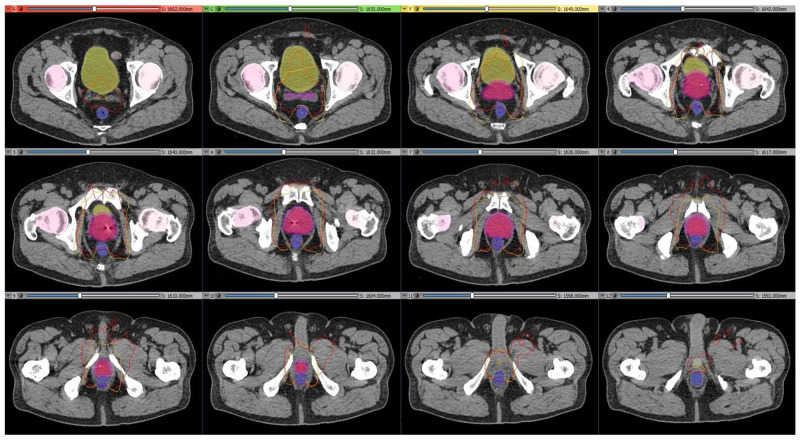
An example of 50% isodose lines. An example of a patient axial images: 50% isodose lines of Halcyon (orange lines) and CK (red lines) plans. Images, structures and isodose lines were generated with 3D Slicer 4.10.2 [24].

The averaged dose volume histograms (DVH) for the targets and critical structures of the CyberKnife and Halcyon plans are given in Figure 3. The Halcyon^TM^ 2.0 is capable of generating plans which satisfy the plan criteria of RTOG 0938 protocol. However, plans have significant dose differences with CyberKnife plans in high dose regions for the targets and low dose regions for the critical organs, except urethra and left femoral head. Those differences in the DVHs are mainly because of the different designs of the two systems. Halcyon^TM^ 2.0 delivers isocentric, coplanar and MLC modulated arc beams; on the other hand, the CyberKnife G4 system delivers non-isocentric, non-coplanar and cone-shaped beams. Several studies have shown that isocentric arc plans modulated with multi-leaf collimators provide excellent dose conformity and coverage of the target [19-22]. VMAT also provides homogeneous plan with high dose gradient [23]. Similar to those studies, Halcyon VMAT plans generated cooler and more conformal plans compared to CK system. There are statistically significant improvements (p < 0.05) on the conformity, Paddick conformity and homogeneity indexes (Figure 1g-1i). The maximum dose to skin and 50% isodose line fall-off are better with VMAT Halcyon plans as well (Figure 4 and Figure 5). Also, there are significant differences between high dose parts of PTV, CTV, urethra and skin DVHs and mean doses of rectum and bladder. Since all these values decreased significantly at VMAT plans, increasing the prescription dose (i.e. 3750 cGy) is an option to increase the mean PTV dose with Halcyon^TM^ 2.0.

Regarding the organs at risk, dose volume histograms show that Halcyon plans improved almost all the critical organ constraints except the left femoral head. The left femoral head dose from CyberKnife plans is lower because of the robotic design of the machine. The CK non-coplanar plans are delivering less dose through the left side of the feet first supine positioned patients. The average values of Halcyon plans are higher for D1cc of the rectum, D1cc and D10% of the bladder constraints. However, there are no statistically significant differences among those values between CK and Halcyon plans (p > 0.05). Also, bladder and rectum mean doses decreased significantly with Halcyon 2.0 plans (Figure 1e and Figure 1f).

In addition to DVH differences, Halcyon^TM^ 2.0 fast arc delivery generated highly conformal plans (Figure 1g and Figure 1h) with a total delivery time of 4.15 ± 0.11 min, excluding setup and imaging time. The CK plans estimated treatment time per fraction is 34 ± 0.5 min, which includes setup and imaging time of about 5 minutes. The CyberKnife system takes two orthogonal kilo-voltage X-rays during the treatments at a selected time interval which affects the treatment time.

Limitation of the study and Halcyon^TM^ 2.0 plan delivery

CK sequential planning is well-known planner experience dependent. The limited number of plans in the study may increase the variation of dosimetric results. It is also reflected from the relatively large standard deviation bars for CK in Figure 1. The newly introduced CK VOLO optimizer may generate plans with less user dependent. In the future, it may worth a further evaluation and comparison for the CK plans with MLC option.

Plans are compared only dosimetrically. However, patient positioning and motion management are still potentially problematic with the Halcyon^TM^ 2.0 system since currently there is no intrafraction monitoring, as with the Varian EDGE/TrueBeam line, and the couch has only three degrees of freedom. The couch has no rotational corrections, and if the position needs to be corrected rotationally, this can be done manually only in the yaw direction in the room. These shortcomings make it especially critical to establish practical pre-treatment prep protocol and to strongly advise the patients to follow the protocol. The patients are to follow the same protocol before the CT sim and each actual treatment delivery, ensuring the ideal patient setup during the actual treatment delivery.

The Halcyon^TM^ 2.0 requires daily imaging before each treatment due to lack of light field and the optical distance indicator. Therefore, CBCT or portal images have to be taken before each treatment. Cai et al. showed that Halcyon 2.0 kV-CBCT with iterative reconstruction satisfies requirements for clinical use [25]. The same study also reported CBCT acquisition time at Halcyon 2.0 is improved and faster compared to the C-arm linac systems. That gives the option of taking a fast kV-CBCT before each or selected beams to verify fiducial or anatomy matching and apply the shifts immediately before the treatment beam. Even though there is no intrafraction tracking with Halcyon 2.0, CBCT provides three-dimensional data of the target and critical organs. The Halcyon 2.0 system at the FROS clinic has Pelvis Fast and Pelvis Large Fast protocols with a scan time of 21.2 s and 25 s, respectively. Both protocols have 2.0 mm thickness. We expect Varian will make the intrafraction tracking with Halcyon available in the near future, which will provide additional safeguarding for the hypo-fractionated SBRT delivery. Until then, an extra CBCT before each arc delivery is warranted.

## Conclusions

This study investigates the potential of SBRT prostate treatment on a Halcyon^TM^ 2.0 unit. The results show that all the re-plans on a HalcyonTM 2.0 meet the criteria of the RTOG 0938 protocol. Dosimetrically, the Halcyon^TM^ 2.0 is capable of generating acceptable SBRT prostate plans based on the RTOG 0938 protocol goals and constraints.
